# Betaine Downregulates RARRES1 to Alleviate Cartilage Fibrosis and Promote Hyaline Cartilage Repair

**DOI:** 10.3390/ijms27156684

**Published:** 2026-07-27

**Authors:** Shiqi Wang, Yang Xue, Jiarui Zhuang, Nuo Xu, Zhaofeng Zhang, Guihua Tan, Huiming Jiang, Rui Wu, Dongquan Shi

**Affiliations:** 1 Laboratory for Bone and Joint Disease, Division of Sports Medicine and Adult Reconstructive Surgery, Department of Orthopedic Surgery, Nanjing Drum Tower Hospital Clinical College of Nanjing University of Chinese Medicine, 321 Zhongshan Road, Nanjing 210008, China; ture406@163.com (S.W.); 17805110988@163.com (Y.X.); zhuang2025@126.com (J.Z.); xxxnisok@163.com (N.X.); 2 Laboratory for Bone and Joint Disease, Division of Sports Medicine and Adult Reconstructive Surgery, Department of Orthopedic Surgery, Nanjing Drum Tower Hospital, Affiliated Hospital of Medical School, Nanjing University, 321 Zhongshan Road, Nanjing 210008, China; 18330586115@163.com (Z.Z.); m18795950086@163.com (R.W.); 3 Laboratory for Bone and Joint Disease, Division of Sports Medicine and Adult Reconstructive Surgery, Department of Orthopedic Surgery, Nanjing Drum Tower Hospital, School of Life Sciences, Nanjing University, 321 Zhongshan Road, Nanjing 210008, China; gwennethgh@163.com; 4 Laboratory for Bone and Joint Disease, Division of Sports Medicine and Adult Reconstructive Surgery, Department of Orthopedic Surgery, Nanjing Drum Tower Hospital Clinical College of Nanjing Medical University, 321 Zhongshan Road, Nanjing 210008, China; jhm15180308@163.com

**Keywords:** cartilage fibrosis, WGCNA, machine learning, betaine

## Abstract

Cartilage degeneration is the hallmark pathological alteration in osteoarthritis (OA). The irreversible accumulation of fibrotic cartilage compromises joint function and accelerates disease progression. However, reliable biomarkers and therapeutic targets for cartilage fibrosis remain lacking. Through bioinformatic analysis of bulk RNA sequencing and single-cell RNA sequencing datasets, RARRES1 (retinoic acid receptor responder 1) was identified as a key biomarker associated with cartilage degeneration. The functional role of RARRES1 was investigated using a CTGF (Connective tissue growth factor) induced chondrocyte fibrosis model and siRNA-mediated gene knockdown. Subsequently, in vitro and in vivo experiments were conducted, including Western blotting, functional assays, flow cytometry, and pathological staining. RARRES1 was markedly upregulated in the damaged cartilage regions of patients with osteoarthritis, and this finding was confirmed in the chondrocyte fibrosis model. Betaine downregulates RARRES1 and promotes hyaline cartilage repair. Importantly, RGS2 was identified as a critical gene through which betaine exerts its effects on scavenging reactive oxygen species (ROS) accumulation. Our study demonstrates that betaine inhibits RARRES1, thereby appearing to upregulate RGS2 and promote ROS clearance to alleviate fibrotic changes in cartilage. RARRES1 may serve as a biomarker and a potential therapeutic target for cartilage fibrosis.

## 1. Introduction

Osteoarthritis (OA) is a degenerative joint disease characterized by anatomical and functional impairment [1]. Its defining pathological features include cartilage degeneration, osteophyte formation, and subchondral bone sclerosis, with cartilage degeneration being the most critical [2]. According to the World Health Organization, OA currently affects more than 500 million people globally, representing a major public health burden [3]. The prevailing clinical view is that OA remains incurable, as the fibrocartilage that forms following injury possesses inferior mechanical properties and often accelerates disease progression [4,5,6]. Consequently, therapeutic strategies that target cartilage and specifically mitigate its fibrosis are central to OA treatment.

In recent years, bioinformatics and machine learning have emerged as essential tools for the systematic investigation of disease [7]. These approaches provide a digital framework for gaining novel insights into gene-level alterations during pathogenesis [8]. Weighted Gene Co-expression Network Analysis (WGCNA) identifies gene modules strongly correlated with clinical phenotypes, and their intersection with disease-specific differentially expressed genes can reveal potential regulatory genes involved in the disease mechanism [9,10].

In this context, our study aimed to analyze disease-related differentially expressed genes from GEO datasets using bioinformatics techniques and to identify significantly associated genes for the disease based on Weighted Gene Co-expression Network Analysis (WGCNA). Subsequently, the Least Absolute Shrinkage and Selection Operator (LASSO), Support Vector Machine-Recursive Feature Elimination (SVM-RFE), and Random Forest (RF) techniques were applied to screen potential candidate genes, and the results were evaluated using logistic regression analysis [11,12]. Despite these methodological advances, the molecular drivers of cartilage fibrosis in OA remain poorly characterized. In this study, we aimed to integrate bulk and single-cell transcriptomic data with machine learning approaches to systematically identify genes associated with cartilage degeneration.

Betaine, chemically known as N,N,N-trimethylglycine, is a typical zwitterionic quaternary ammonium compound [13,14]. In recent years, numerous studies have demonstrated that betaine exerts significant protective effects in the progression of various fibrotic diseases [15,16,17], effectively attenuating ECM deposition and inhibiting tissue remodeling [18]. However, the downstream key effector molecules and the precise signal transduction networks mediating the aforementioned antifibrotic protective effects of betaine have not yet been systematically elucidated and require further in-depth investigation.

Single-cell sequencing is a next-generation sequencing technology that has emerged in recent years, enabling the exploration of gene expression heterogeneity at the individual cell level. Single-cell virtual knockout is a recently developed algorithm based on single-cell sequencing data, used for predicting the potential mechanisms of genes. By integrating OA cartilage samples from the GEO dataset, we identified the localization of RARRES1 in the single-cell data and found that it exhibits strong heterogeneity with markers of the chondrogenic phenotype (COL2A1, SOX9, ACAN). Further single-cell virtual knockout was employed to untangle the potential mechanism by which RARRES1 influences cartilage fibrosis. To further validate the function of this gene and investigate whether betaine exerts therapeutic effects by regulating RARRES1, we conducted a series of experimental validations. An in vitro chondrocyte fibrosis model was employed to assess the changes in RARRES1 and RGS2. Additionally, an in vivo cartilage repair model was established to test whether betaine inhibits RARRES1 and thereby ameliorates cartilage fibrotic repair. Finally, in vitro experiments were performed to explore the potential mechanism by which betaine suppresses RARRES1 to promote cartilage repair.

In summary, this study integrates bioinformatics approaches with molecular functional validation to identify and characterize RARRES1 as a novel gene closely associated with cartilage fibrotic changes. These findings provide new molecular insights into the pathogenesis of OA and may contribute to the improvement of early diagnosis and therapeutic strategies for OA.

## 2. Results

### 2.1. WGCNA and Differential Gene Analysis Yielded Six Intersecting Genes

**Figure 1:**
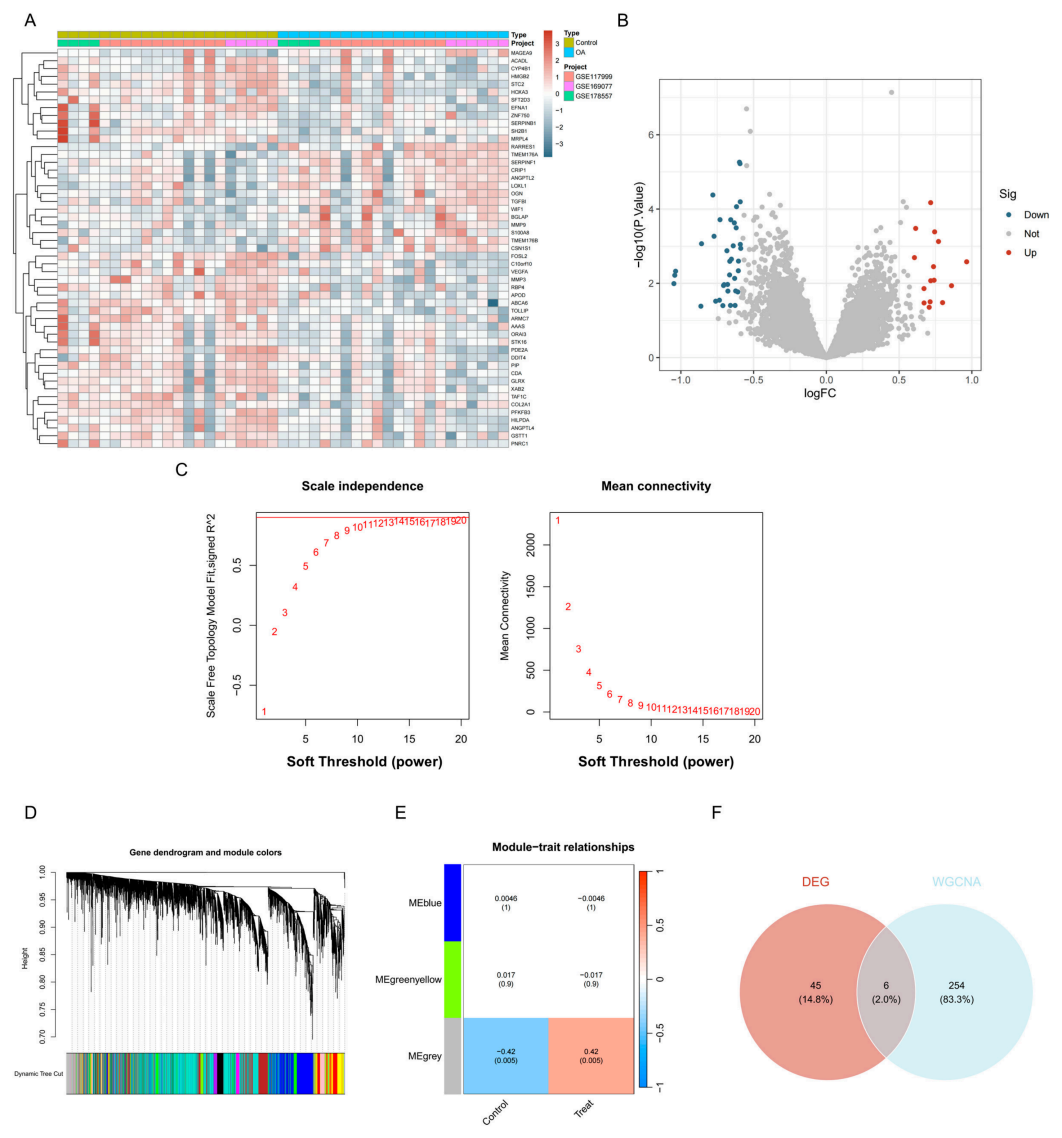
Weighted gene co-expression network analysis (WGCNA) and identification of differentially expressed genes (DEGs). (**A**) Heatmap of DEGs from integrated cartilage transcriptome datasets; (**B**) Volcano plot of DEGs from integrated cartilage transcriptome datasets, red indicates significantly upregulated genes, while blue indicates significantly downregulated genes; (**C**) Scale-free network co-expression matrix; (**D**) Dendrogram of gene module selection in WGCNA, the colors represent the identified gene modules; (**E**) Heatmap of module-trait association analysis; (**F**) Venn diagram depicting the overlap between WGCNA and DEGs.

All datasets were normalized to eliminate systematic sample errors. This normalization enhanced data homogeneity, supporting subsequent analyses (Supplementary Figure S1A,B). Using established screening criteria, a total of 51 differentially expressed genes (DEGs) were identified, comprising 15 upregulated and 36 downregulated genes. These DEGs were visualized using a volcano plot and a heatmap (Figure 1A,B). The optimal soft threshold was selected as 12, corresponding to the minimum parameter that satisfies a scale-free topology fit index (R^2^) greater than 0.85 (Figure 1C). Based on the correlation structure among samples, a preliminary hierarchical clustering analysis was performed (Figure 1D), and the data were subsequently divided into three distinct modules. Among these, the grey module showed the most significant association with the disease phenotype (*p* = 0.005) (Figure 1E). An intersection analysis between the genes in this module and the set of DEGs identified six candidate genes (Figure 1F): RARRES1 (retinoic acid receptor responder 1), C10orf10 (chromosome 10 open reading frame 10), CSN1S1 (alpha-S1-casein), MMP3 (matrix metalloproteinase 3), APOD (apolipoprotein D), and VEGFA (vascular endothelial growth factor A).

### 2.2. Machine Learning and Univariate Logistic Regression Analysis Identified RARRES1 as the Gene Most Closely Associated with Cartilage Degeneration

**Figure 2:**
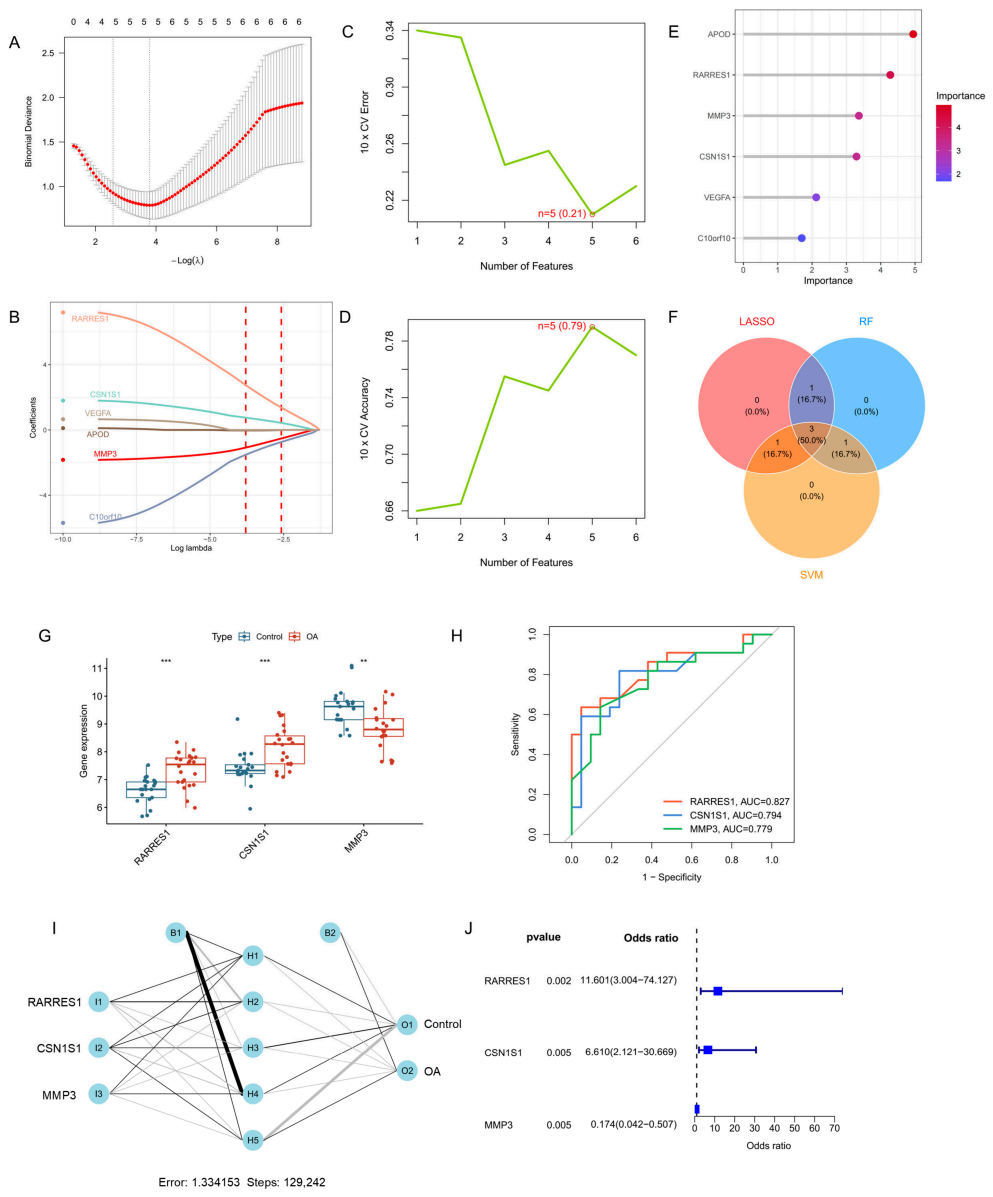
Machine learning and univariate logistic regression analysis. (**A**,**B**) Least absolute shrinkage and selection operator (LASSO) regression identifies biomarkers of cartilage degeneration; (**C**,**D**) Support Vector Machine-Recursive Feature Elimination (SVM-RFE) identifies biomarkers of cartilage degeneration; (**E**) RandomForest (RF) identifies biomarkers of cartilage degeneration; (**F**) Venn diagram of cartilage degeneration biomarkers identified by three machine learning; (**G**) Expression levels of three candidate core genes; (**H**) The Area under the curve (AUC) values of the three candidate genes in the dataset; (**I**) Visualization of the ANN diagnostic model, B2 is connected to the OA group by a black solid line; (**J**) Visualization of logistic regression tree diagram. ns: not significant, ** *p* < 0.01 and *** *p* < 0.001.

To more accurately identify disease-associated genes, we applied three machine learning algorithms: LASSO regression, SVM-RFE, and RF to further screen the six intersecting genes obtained from WGCNA and differential expression analysis. LASSO regression selected five genes: RARRES1, C10orf10, CSN1S1, MMP3, and APOD (Figure 2A,B). SVM-RFE determined the optimal feature number as five, corresponding to the lowest model complexity and overfitting risk; the five genes identified were RARRES1, C10orf10, MMP3, VEGFA, and CSN1S1 (Figure 2C,D). The random forest algorithm ranked gene importance, and five genes with a contribution score greater than 2 were retained: APOD, RARRES1, MMP3, CSN1S1, and VEGFA (Figure 2E). The intersection of the optimal genes from the three machine learning methods yielded three candidate genes: RARRES1, CSN1S1, and MMP3 (Figure 2F). A neural network model constructed based on these three genes accurately discriminated disease characteristics (Figure 2I). Furthermore, in the OA dataset, RARRES1 and CSN1S1 were significantly upregulated (*p* < 0.001), whereas MMP3 was significantly downregulated (*p* < 0.01) (Figure 2G). All three genes showed high diagnostic sensitivity in the test dataset, with RARRES1 achieving the best accuracy, with an area under the curve (AUC) = 0.827 (Figure 2H). Univariate logistic regression analysis, used to evaluate the independent effect of each variable on the overall outcome, indicated that RARRES1 contributed the most to the overall prediction of the disease (Figure 2J). In the external validation dataset GSE114007, RARRES1 exhibited the most pronounced differential expression among the three candidate genes (Supplementary Figure S4A), with an AUC of 0.944, which was substantially higher than those of CSN1S1 (AUC = 0.583) and MMP3 (AUC = 0.736) (Supplementary Figure S4B). Based on these findings, RARRES1 was selected for subsequent analyses.

### 2.3. RARRES1 Is Significantly Upregulated in Damaged Cartilage and May Mediate Oxidative Stress in the Lesion Area

**Figure 3:**
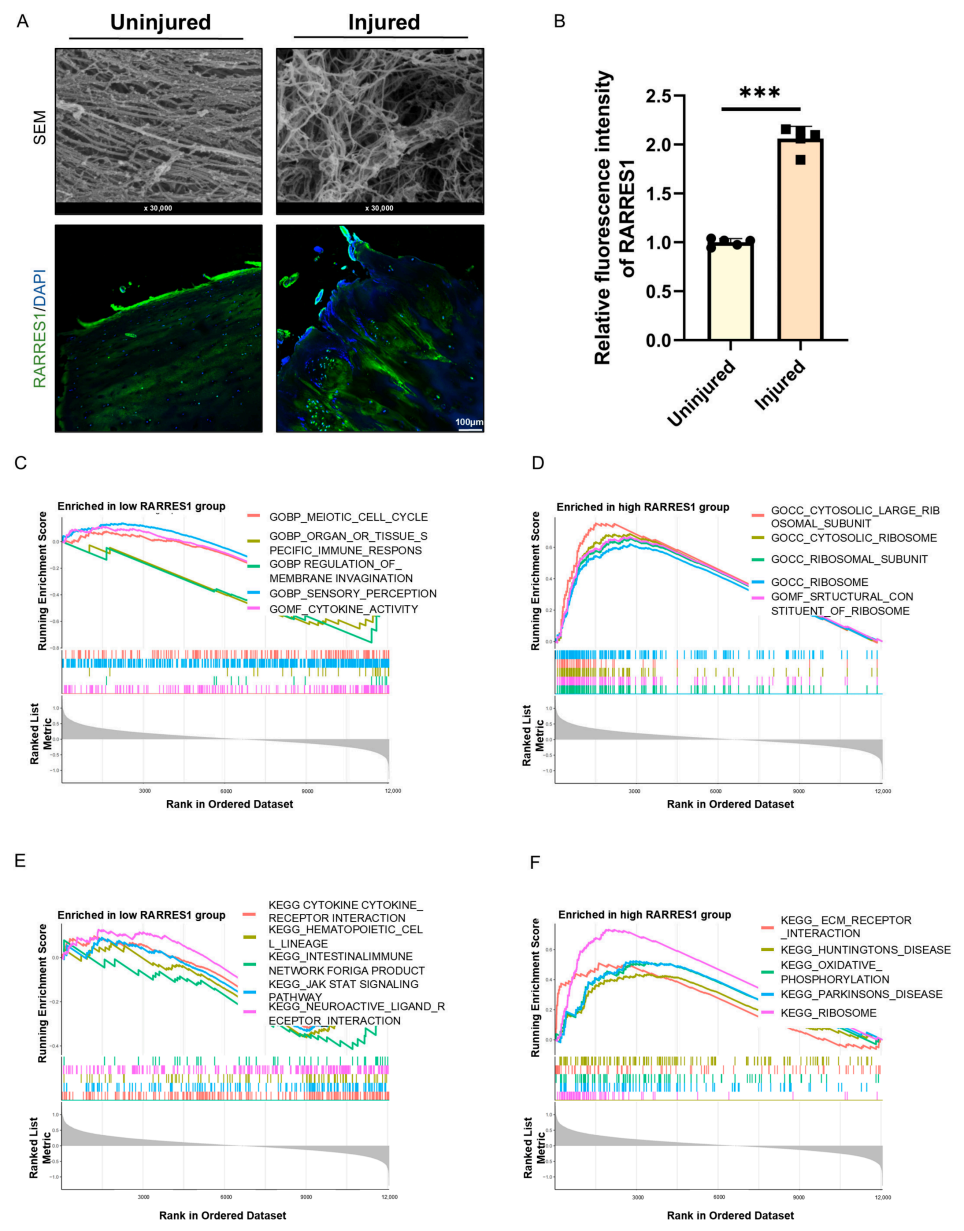
The correlation between Retinoic Acid Receptor Responder Protein 1 (RARRES1) and cartilage degeneration has been validated in clinical samples. (**A**) Scanning electron microscopy (SEM) of clinical specimens and RARRES1 immunofluorescence staining (SEM, *n* = 1; IF, *n* = 5, scale bar = 100 μm), collagen fibers were orderly arranged in the non-injured area, whereas disorganized collagen arrangement was observed in the injured area; (**B**) Comparative analysis of relative fluorescence intensity of RARRES1, *** *p* < 0.001; (**C**) Gene ontology (GO) pathways enriched in the RARRES1 low-expressing subset; (**D**) GO pathways enriched in the RARRES1 high-expressing subset; (**E**) Kyoto Encyclopedia of Genes and Genomes (KEGG) pathways enriched in the RARRES1 low-expressing subset; (**F**) KEGG pathways enriched in the RARRES1 high-expressing subset.

To determine the expression changes of RARRES1 in OA cartilage degeneration, we collected clinical samples and performed immunofluorescence staining of RARRES1 in lesional and non-lesional areas. The results showed that RARRES1 expression was significantly upregulated in the lesional area (Figure 3A,B). To further explore the function of RARRES1, we conducted single-gene GSEA analysis to evaluate the impact of RARRES1 perturbation on the entire gene set. The GSEA results based on GO terms revealed that in the RARRES1 low-expression group, terms such as cell cycle and cytokine activity were significantly enriched (Figure 3C,E); corresponding KEGG pathway analysis also showed enrichment of cytokine–cytokine receptor interaction pathways. In the RARRES1 high-expression group, GO terms related to the Golgi apparatus and ribosomes were significantly enriched, while the corresponding KEGG pathways showed significant enrichment of ECM interaction and oxidative stress-related pathways (Figure 3D,F). These findings indicate that RARRES1 expression is low in homeostatic chondrocytes, and its upregulation suggests that chondrocytes may actively engage in ECM-related synthesis while being under oxidative stress.

### 2.4. Single-Cell Virtual Knockout Predicts That RARRES1 Promotes Upregulation of RGS2

**Figure 4:**
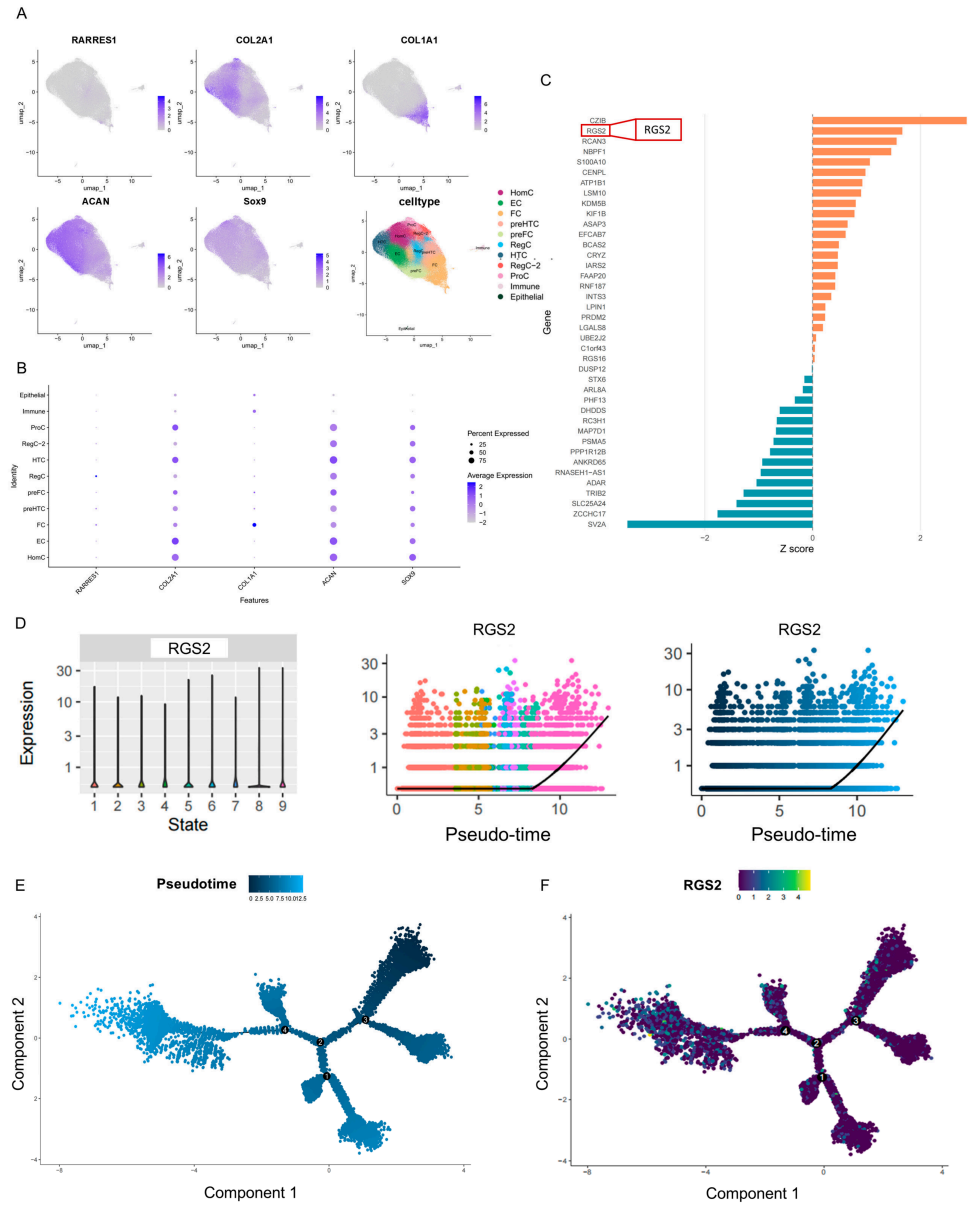
Single-cell virtual knockout predicts that RARRES1 promotes the upregulation of Regulator of G Protein Signaling 2 (RGS2). (**A**) Uniform Manifold Approximation and Projection (UMAP) plots showing the expression of RARRES1, Collagen Type II Alpha 1 Chain (COL2A1), Collagen Type I Alpha 1 Chain (COL1A1), SRY-box Transcription Factor 9 (SOX9), and Aggrecan (ACAN) in Osteoarthritis (OA) cartilage subpopulations; (**B**) Bubble plots depicting the expression of RARRES1, COL2A1, COL1A1, SOX9, and ACAN across different subpopulations; (**C**) Gene perturbation results following the virtual knock-out of RARRES1; (**D**) Expression level of RGS2 in the differentiation trajectory of the Regulatory Chondrocyte (RegC) subpopulation; (**E**) Differentiation trajectory of RGS2 in RegC; (**F**) RGS2 expression dynamics along the differentiation trajectory of the RegC subpopulation.

To further elucidate the cellular localization of RARRES1, we analyzed two OA cartilage single-cell datasets, GSE255460 and GSE220243, to perform subpopulation localization of RARRES1, the signature genes of the cartilage subpopulations are presented in Supplementary Figure S2. Although RARRES1 expression levels were low, we identified a relatively specific RegC subpopulation for RARRES1 (Figure 4B). COL1A1, COL2A1, SOX9, and ACAN are important markers for evaluating cartilage fibrosis; the localization of these genes in the UMAP plot revealed heterogeneity between the FC (fibrocartilage) subpopulation and other cartilage subpopulations (Figure 4A). The virtual knockout algorithm is a bioinformatics method that predicts the functional consequences of knocking out a specific gene on cellular functions. Virtual knockout analysis targeting RARRES1 showed a significant upregulation of RGS2, suggesting that RGS2 may be a key downstream target of RARRES1 (Figure 4C). Monocle2 divides trajectories into distinct segments based on branches, with each state representing a stage in the developmental process. Pseudotime analysis of the RegC subpopulation indicated that RGS2 was upregulated at the terminal stage of development within this subpopulation (Figure 4D). The differentiation trajectory of all RegC subpopulation cells showed four potential branches, where dark blue represents early development and light blue represents late development. Focusing on RGS2, the cell differentiation trajectory likewise revealed upregulated expression in late differentiation (Figure 4E). Given that RGS2 is a known inhibitor of tissue fibrosis, the RegC subpopulation may be critically involved in suppressing cartilage fibrosis [19,20,21].

### 2.5. Betaine Is a Potential Therapeutic Agent Targeting RARRES1

**Figure 5:**
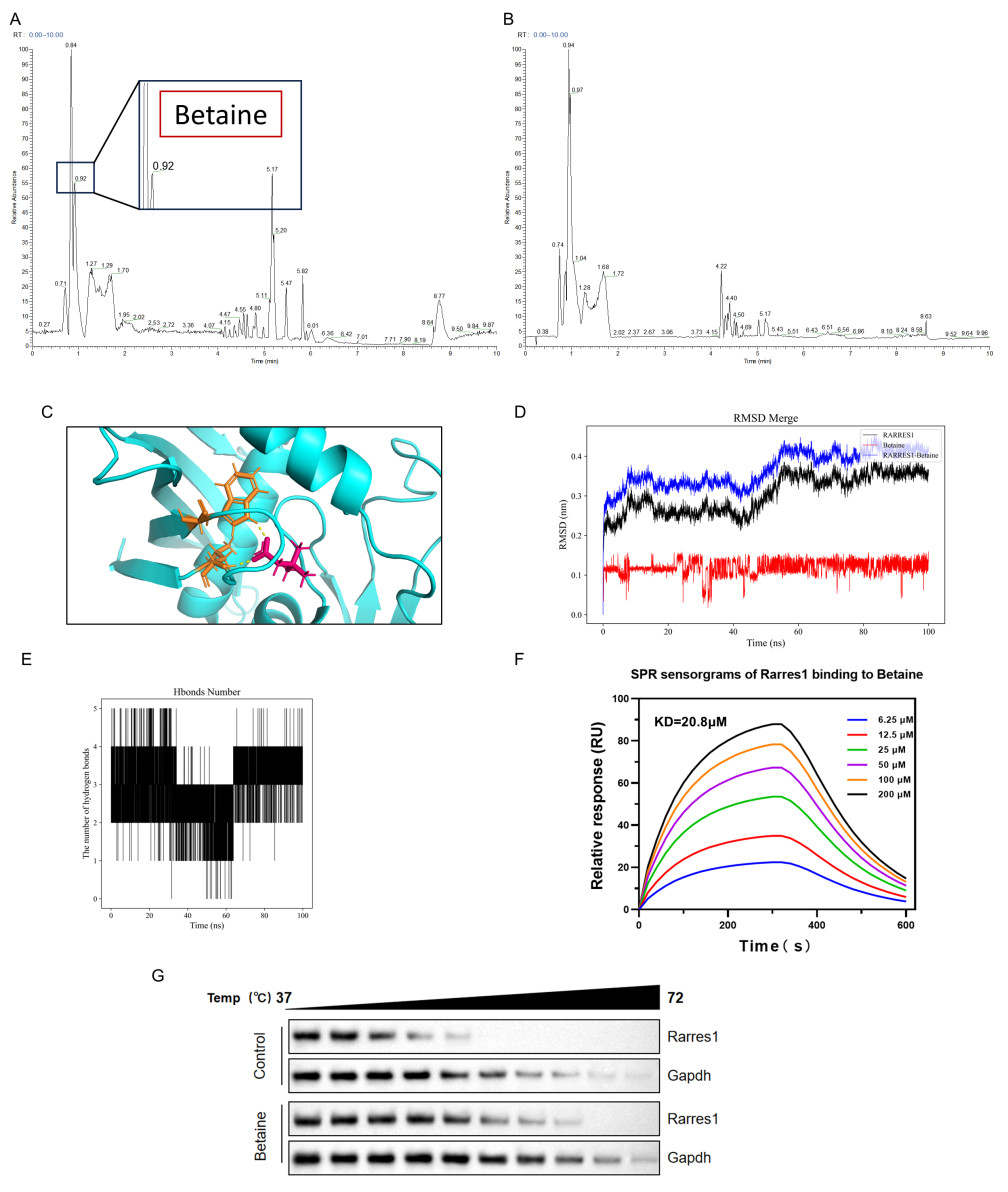
Betaine is a potential therapeutic agent targeting RARRES1. (**A**) Metabolite peak map in positive ion mode; (**B**) Metabolite peak map in negative ion mode; (**C**) Schematic diagram of molecular docking between betaine and RARRES1; (**D**) Root-Mean-Square Deviation (RMSD) Plot for Betaine Binding to RARRES1 (RUN1); (**E**) Number of Hydrogen Bonds in Betaine-RARRES1 Binding (RUN1); (**F**) Surface plasmon resonance (SPR) sensorgrams showing the response curves obtained when different concentrations of betaine (6.25, 12.5, 25, 50, 100, and 200 μM) were flowed over the chip surface with immobilized RARRES1. (**G**) 400 µM betaine significantly enhances the thermal stability of the RARRES1 protein. (Temperature gradients were set at 37, 40, 42, 45, 47, 52, 57, 62, 67, and 72 °C).

To screen for bioactive compounds that may modulate RARRES1 expression, we performed chromatography-mass spectrometry on CXTB capsules from the Supplementary Table S1. Among all metabolites detected in both positive and negative ionization modes, betaine exhibited the highest peak intensity among druggable natural compounds. Among the annotated features, arginine showed the highest peak intensity; however, arginine is a ubiquitous endogenous amino acid widely distributed in biological matrices and is not a unique constituent of CXTB. The second most intense peak could not be reliably identified with the available spectral libraries. Therefore, betaine (HMDB ID: HMDB0000267), which ranked third in peak intensity and was unequivocally identified, was selected for subsequent investigation (Figure 5A,B). Its retention time was approximately 0.92 min, the protonated precursor ion ([M+H]^+^) was detected at *m*/*z* 118.0863 (Supplementary Table S1), and the characteristic ion pair of betaine was observed at 118.08 > 59.07 (Supplementary Figure S6). Given that the anti-fibrotic effects of betaine have been well documented in previous studies [15,16,17], it was chosen for further analysis.

To explore whether betaine physically interacts with RARRES1, we performed molecular docking and molecular dynamics simulations. Betaine exhibited potential binding affinity for RARRES1 in molecular docking, with a predicted ΔG of −4.7 kcal/mol (Figure 5C). Based on a comprehensive analysis of three independent 100 ns molecular dynamics simulations, this study confirms the formation of a stable complex system between Betaine and the RARRES1 protein, characterized by favorable binding affinity. The radius of gyration results indicate that the protein maintains a highly compact folded state upon drug binding. Hydrogen bond analysis reveals that approximately three hydrogen bonds are stably maintained between the drug and the protein. RMSD analysis demonstrates excellent structural stability for both the protein backbone and the drug molecule, with the drug exhibiting an extremely low RMSD (approximately 0.11–0.12 nm), indicating its well-defined localization and stable orientation within the binding pocket. RMSF analysis unveils a reasonable dynamic profile, characterized by overall structural rigidity of the protein along with moderate local flexibility. MM/GBSA binding free energy calculations further energetically confirm the spontaneous binding trend between the drug and the target protein. The total binding free energies across the three simulations are consistently in the range of −16 to −17 kcal/mol, with van der Waals and electrostatic interactions serving as the primary driving forces. The high degree of consistency among the three independent simulation results robustly validates the stability and reliability of the simulated system, demonstrating that Betaine, as a bioactive modulator of RARRES1, possesses favorable structural stability and molecular binding characteristics. (Supplementary Figure S5) (Supplementary Table S2). To experimentally validate these computational predictions, we performed surface plasmon resonance (SPR) and cellular thermal shift assay (CETSA). SPR analysis revealed that betaine bound to RARRES1 in a concentration-dependent manner, with the binding curves fitting well to a 1:1 Langmuir binding model (Figure 5F). CETSA further demonstrated that betaine (400 µM) significantly increased the thermal stability of RARRES1 (Figure 5G). The above results confirmed that betaine directly binds to recombinant human RARRES1.

### 2.6. Betaine Reverses the Upregulation of RARRES1 and Promotes RGS2 Expression in Chondrocyte Fibrosis

**Figure 6:**
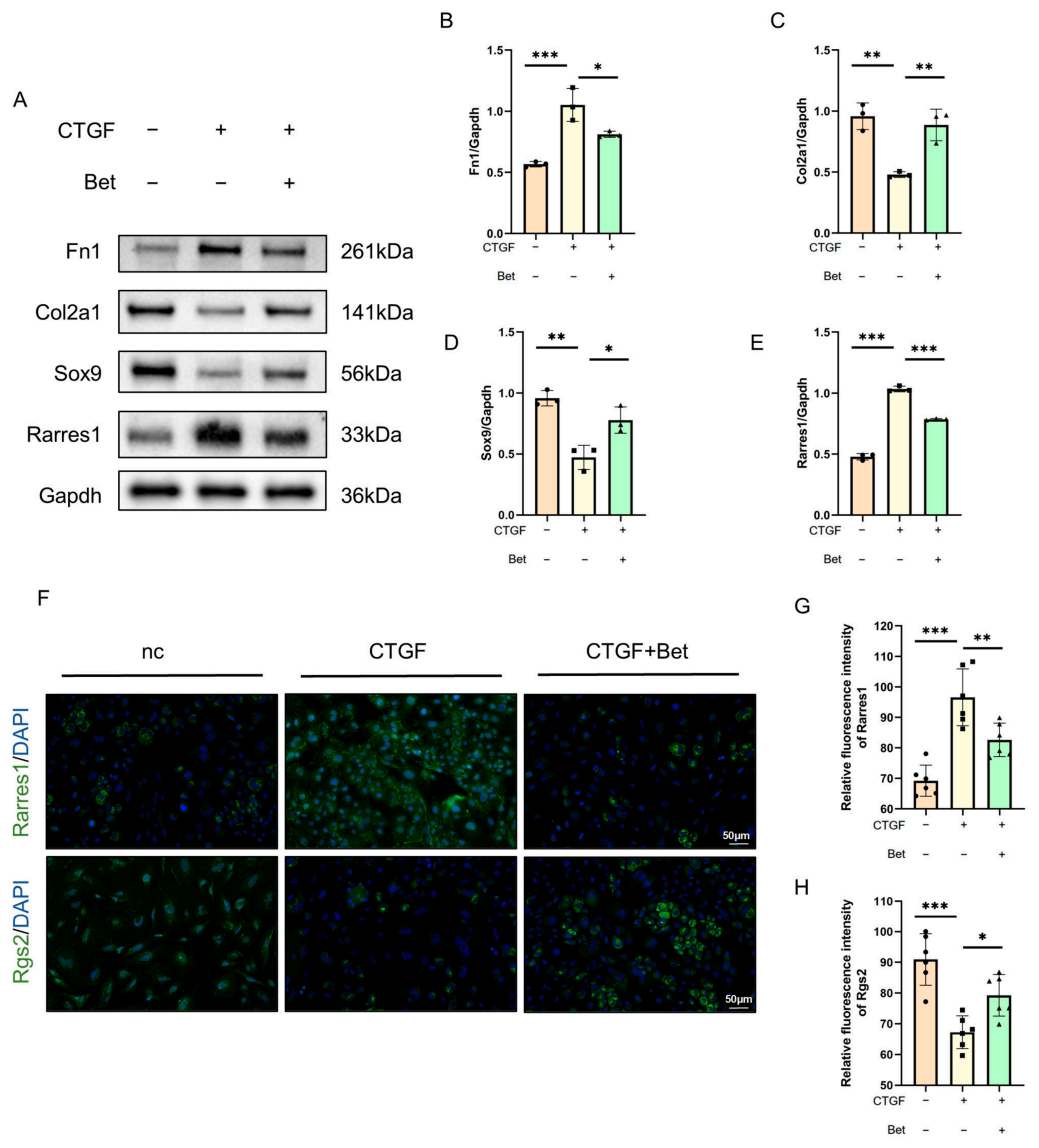
Betaine reverses the upregulation of Rarres1 and promotes Rgs2 expression in chondrocyte fibrosis. (**A**) Western blot analysis of Fibronectin 1 (Fn1), Col2a1, Sox9, and Rarres1 in the chondrocyte fibrosis model following betaine treatment; (**B**–**E**) Quantitative analysis of Fn1, Col2a1, Sox9, and Rarres1 expression in vitro (*n* = 3); (**F**) Staining of Rarres1 and Rgs2 in the chondrocyte fibrosis model (*n* = 6, scale bar = 50 μm); (**G**,**H**) Comparative analysis of relative fluorescence intensity of Rarres1 and Rgs2. ns: not significant, * *p* < 0.05, ** *p* < 0.01, and *** *p* < 0.001.

To further validate whether RARRES1 can serve as a biomarker for fibrotic alterations in cartilage and to assess the effect of betaine on RARRES1 at the protein level, experiments were conducted using rat primary chondrocytes in vitro (Figure 6A). CTGF, a core growth and healing signal, plays a role in promoting fibrotic changes during the tissue healing process. Our results demonstrated that in CTGF-induced rat primary chondrocytes, Fn1 was upregulated, while Col2a1 and Sox9 were both suppressed, indicating successful induction of chondrocyte fibrosis by CTGF (Figure 6B–D). In this context, Rarres1 was upregulated, suggesting that RARRES1 can serve as a marker of chondrocyte fibrosis (Figure 6E). Following the application of betaine in conjunction with CTGF, the fibrotic indicators were alleviated, and RARRES1 expression was also significantly inhibited. Immunofluorescence analysis further confirmed that RARRES1 was upregulated in the chondrocyte fibrosis model and downregulated after betaine treatment (Figure 6B–E). Additionally, RGS2 expression was downregulated in the cartilage fibrosis model and was restored following betaine treatment (Figure 6F–H). These findings suggest that betaine may reverse the upregulation of RARRES1 and improve RGS2 expression in chondrocyte fibrosis.

### 2.7. Betaine Upregulates RGS2 and Alleviates Reactive Oxygen Species Accumulation

**Figure 7:**
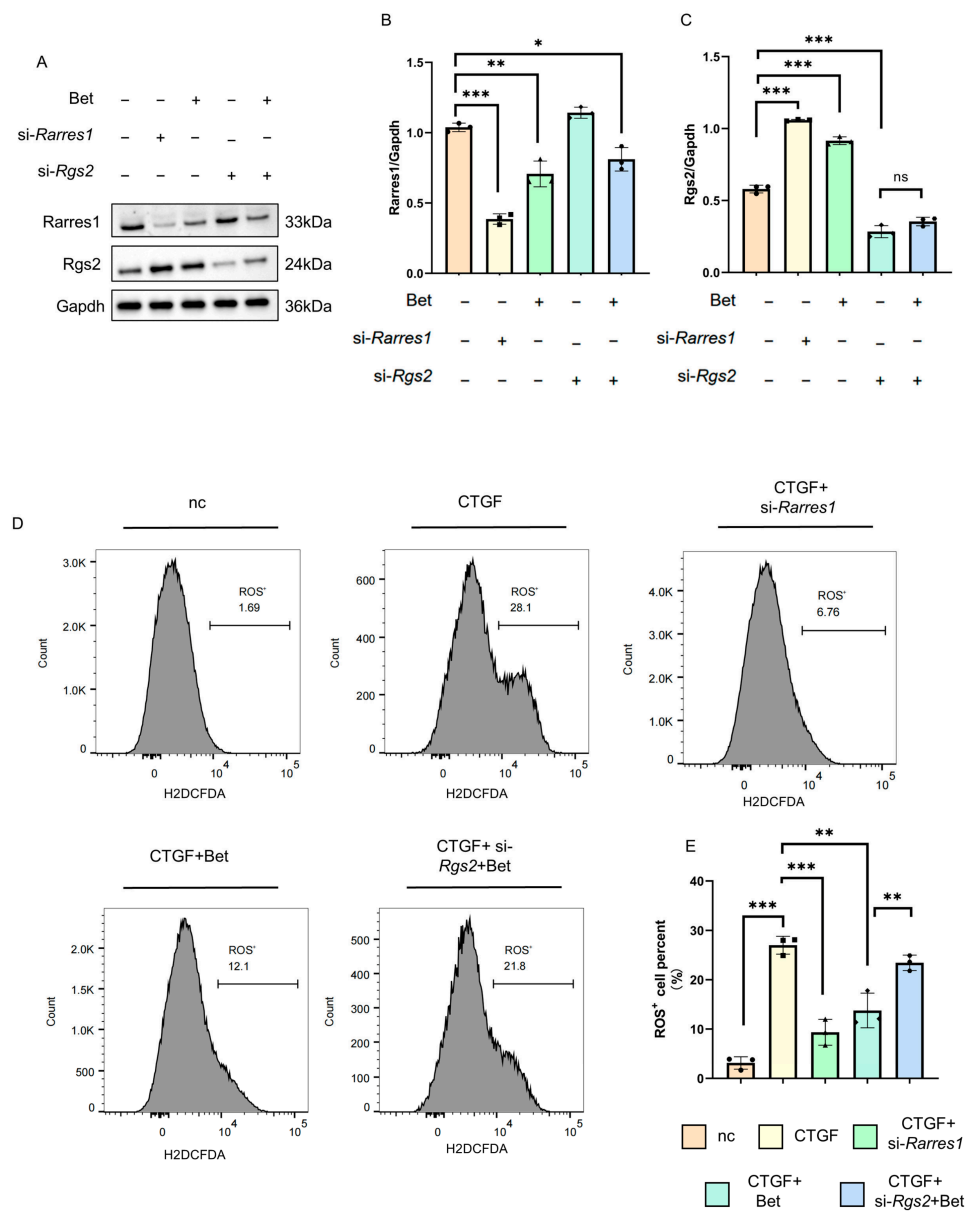
Betaine inhibits Rarres1 and alleviates Reactive Oxygen Species (ROS) accumulation. (**A**) Betaine inhibits Rarres1 and upregulates Rgs2; (**B**,**C**) Quantitative analysis of Rarres1 and Rgs2 expression in vitro (*n* = 3); (**D**) Betaine inhibits ROS accumulation in the Connective Tissue Growth Factor (CTGF) induced cartilage fibrosis model (*n* = 3); (**E**) Quantification of ROS^+^ cell numbers. ns: not significant, * *p* < 0.05, ** *p* < 0.01, and *** *p* < 0.001.

Previous results suggested that RARRES1 may negatively regulate RGS2. Therefore, we employed siRNA technology to knock down RARRES1 and RGS2, respectively. The results indicated that RARRES1 knockdown promoted RGS2 expression, whereas RGS2 knockdown did not significantly affect RARRES1 expression, suggesting that RGS2 likely functions downstream of RARRES1 (Figure 7A). Betaine treatment alone reduced RARRES1 expression and was accompanied by an upregulation of RGS2 in vitro; however, betaine did not rescue the decrease in RGS2 caused by RGS2 knockdown, suggesting that RGS2 is a critical mediator of the therapeutic effect of betaine (Figure 7B,C). Given that previous studies indicated that RARRES1 upregulation may mediate oxidative stress, we designed in vitro experiments to investigate whether the fibrotic model induces reactive oxygen species (ROS) accumulation via RARRES1. The results revealed that the ROS level was significantly increased in the chondrocyte fibrosis model, and RARRES1 knockdown significantly alleviated ROS accumulation. Betaine also reduced ROS accumulation during chondrocyte fibrosis, and this effect was abolished upon RGS2 knockdown, indicating that RGS2 is a key target through which betaine exerts its ROS-scavenging function (Figure 7D,E).

### 2.8. Betaine Inhibits RARRES1 and Promotes Hyaline Cartilage Repair In Vivo

**Figure 8:**
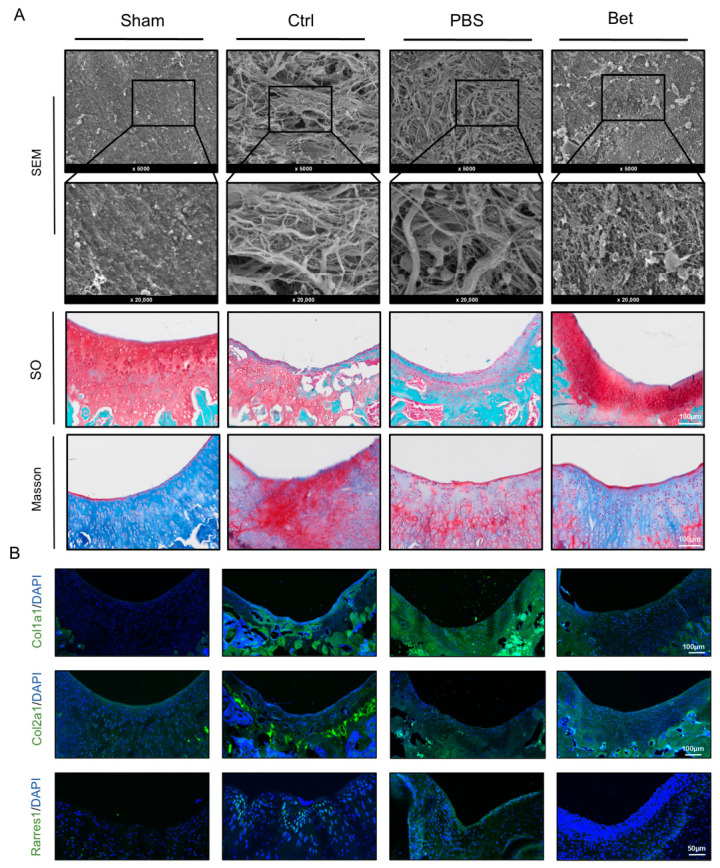

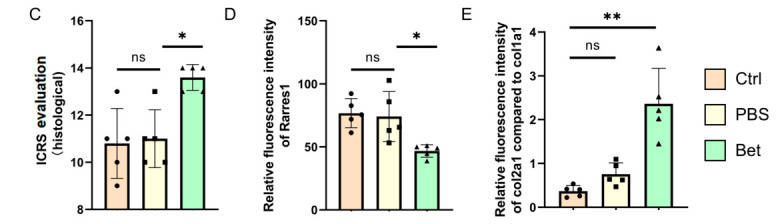
Betaine inhibits Rarres1 and promotes hyaline cartilage repair in vivo. (**A**) Evaluation of Cartilage Repair Using SEM (*n* = 1), Safranin O (SO) (*n* = 5, scale bar = 100 μm) and Masson’s Staining (*n* = 5 scale bar = 100 μm); (**B**) Immunofluorescence staining of Col2a1, Col1a1, and Rarres1 in each group (*n* = 5, scale bar = 100 μm/scale bar = 50 μm); (**C**) International Cartilage Repair Society (ICRS) scores in each group; (**D**) Comparative analysis of relative fluorescence intensity of Rarres1. (**E**) Comparative analysis of relative fluorescence intensity between Col2a1 and Col1a1. ns: not significant, * *p* < 0.05 and ** *p* < 0.01.

For the in vivo study, we established a rat cartilage defect model to evaluate the effect of betaine on hyaline cartilage repair. Scanning electron microscopy revealed that at 8 weeks post-injury, the Ctrl and PBS groups exhibited disorganized fibrous arrangement and reduced ECM deposition on the rat cartilage surface; these alterations were markedly ameliorated in the betaine-treated group. The ICRS (International Cartilage Repair Society) score was used to assess cartilage repair, and the results demonstrated that the betaine group (Bet group) had a significantly higher score than the PBS and Ctrl groups (Figure 8A,C). The ratio of type II collagen to type I collagen (COL2A1/COL1A1) is recognized as the gold standard for evaluating hyaline cartilage repair; betaine treatment significantly increased the COL2A1/COL1A1 ratio in the regenerated cartilage (Figure 8B,E). Collectively, these results demonstrate that betaine promotes hyaline cartilage repair in vivo. Furthermore, tissue fluorescence staining showed that betaine inhibited RARRES1 expression during cartilage fibrotic repair (Figure 8D).

## 3. Discussion

As the joint’s primary load-bearing interface, cartilage distributes loads uniformly, absorbs and dissipates impact forces from movement, and protects the knee from injury [22,23,24,25]. The smooth surface of hyaline cartilage ensures minimal friction during joint articulation [26]. However, cartilage has a limited intrinsic capacity for repair due to its lack of neural and vascular supply, which often leads to the formation of fibrocartilage after injury [27]. Fibrocartilage exhibits substantially reduced elasticity, impairing its ability to mitigate mechanical stress [28,29]. This allows impact forces to transmit directly to the subchondral bone, accelerating osteoarthritis progression [30]. Furthermore, fibrocartilage is more susceptible to wear, and cartilage fragments shed into the joint cavity can readily initiate an inflammatory cascade [31]. Identifying relevant biomarkers and therapeutic targets within cartilage tissue is therefore essential.

As a membrane-associated protein closely linked to the retinoic acid signaling pathway, RARRES1 plays a profound role in regulating cell proliferation, differentiation, apoptosis, and metabolism and has been implicated in various pathological processes. However, its biological function and underlying molecular regulatory mechanisms in cartilage degeneration and repair remain poorly understood. Bioinformatically, WGCNA and machine learning algorithms identified RARRES1 as a top-ranked gene associated with cartilage degeneration. To experimentally validate this association, we examined RARRES1 expression in human OA cartilage and in a CTGF-induced chondrocyte fibrosis model. The consistent upregulation of RARRES1 in both settings confirmed its close correlation with fibrotic changes. Based on these convergent lines of evidence, we conclude that RARRES1 may serve as a biomarker of cartilage fibrosis.

Betaine, a natural methyl donor and osmoprotectant, has been reported to maintain cellular homeostasis and mitigate oxidative stress in various pathological contexts [32,33]. Our in vivo experiments extend these findings by demonstrating that betaine suppresses RARRES1 expression and promotes hyaline cartilage repair in a rat cartilage defect model. This is consistent with recent evidence that betaine exerts geroprotective effects through systemic metabolic regulation [32], yet its specific targeting of RARRES1 in cartilage represents a previously unrecognized mechanism.

**Figure 9:**
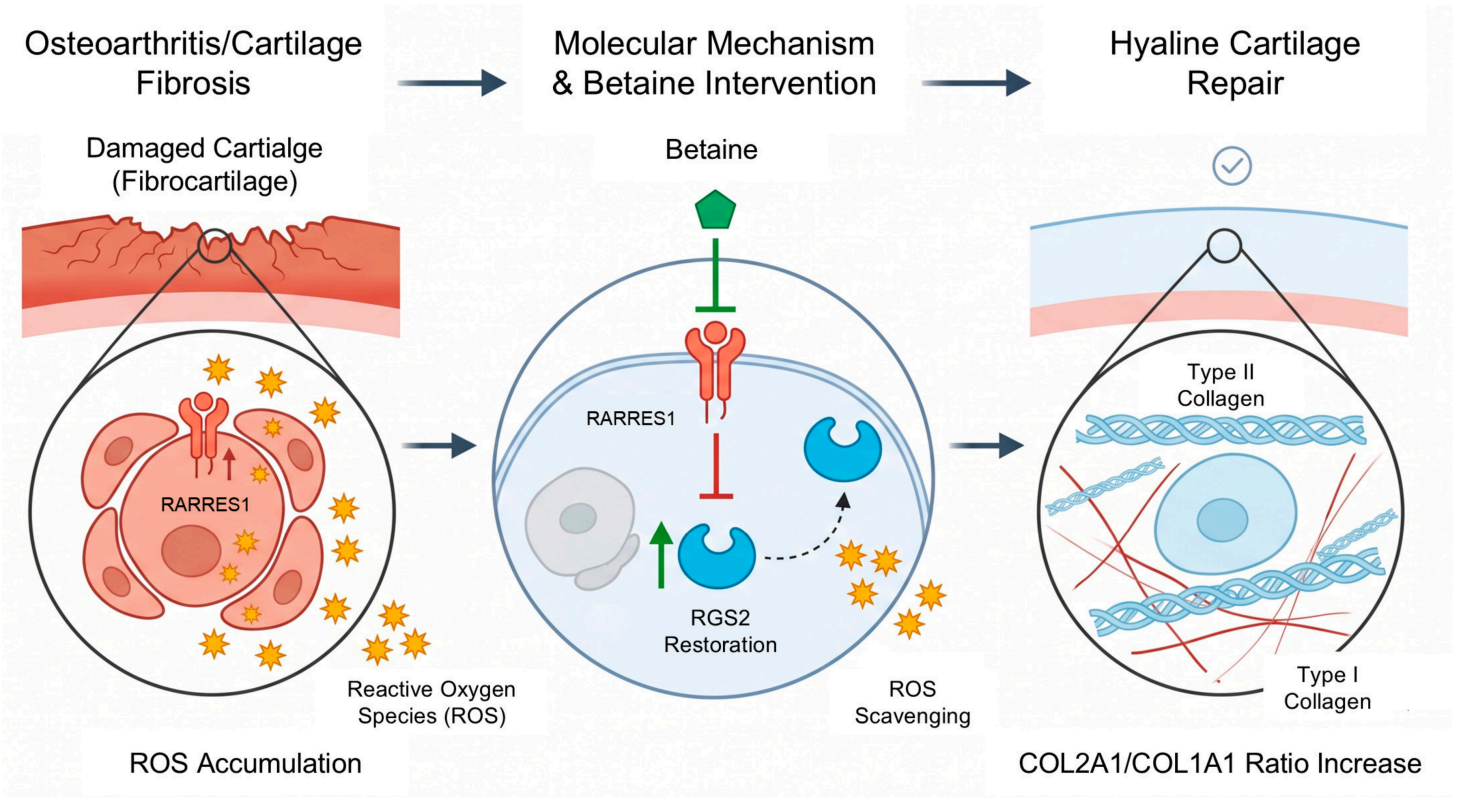
Betaine downregulates RARRES1 to promote hyaline cartilage repair. (Created in BioRender. Wang, S. (2026) https://BioRender.com/0olq826) (accessed on 1 May 2026). Betaine suppresses RARRES1, thereby upregulating RGS2 to enhance ROS clearance and increase the COL2A1/COL1A1 ratio, shifting repair from fibrocartilage toward hyaline cartilage. Created in BioRender style.

We observed that betaine suppressed the expression of RARRES1 at the transcriptional level, and this inhibitory effect effectively antagonized the upregulation of *Rarres1* mRNA induced by CTGF (Supplementary Figure S3), suggesting that transcriptional regulation may be one of the pathways through which betaine downregulates RARRES1 protein expression. The computational docking and MD simulations predicted a potential binding mode between betaine and RARRES1, which was subsequently confirmed by SPR and CETSA experiments. Although betaine targets RARRES1, whether this binding triggers specific post-translational modifications requires further experimental validation. In silico GSEA initially suggested that RARRES1 upregulation might be linked to oxidative stress pathways (Figure 3F). Experimentally, we observed that *Rarres1* knockdown significantly reduced ROS accumulation, while RGS2 knockdown abolished the ROS-scavenging effect of betaine (Figure 7D,E). These experimental data support a model in which betaine, by suppressing RARRES1, relieves its negative regulation of RGS2, thereby enhancing ROS clearance [34,35] and shifting the repair process toward a hyaline cartilage phenotype (Figure 9). Although our findings are consistent with this working model, direct evidence of the RGS2-mediated ROS regulatory machinery warrants further investigation.

Despite the aforementioned findings, this study has several limitations. For instance, we did not investigate whether overexpression of RARRES1 leads to downregulation of RGS2 expression. Instead, through fibrosis models and RARRES1 knockdown experiments, we observed that RARRES1 appears to negatively regulate RGS2 expression. Previous studies on renal, hepatic, and pulmonary fibrosis have emphasized that RGS2 inhibits fibrotic progression by modulating Gq protein activity—a mechanism that was not further explored in the present study. Additionally, although RGS2 has been identified as a key gene in betaine-mediated ROS scavenging, the specific mechanism underlying the relationship between RGS2 and ROS elimination remains to be elucidated. We acknowledge that the exclusive use of male animals in this study represents a potential limitation. Although this sex selection is commonly adopted in experimental osteoarthritis-related disease models to minimize confounding effects of the estrous cycle, it nevertheless limits the generalizability of our findings to female subjects. Given that sex hormones are known to influence chondrocyte metabolism, inflammatory responses, and fibrotic repair processes, future investigations are warranted to systematically evaluate the potential sex-dependent effects on RARRES1 expression and the therapeutic efficacy of betaine, thereby confirming the translational relevance of our observations across sexes.

## 4. Materials and Methods

### 4.1. Acquisition of GEO Data and Analysis of Differentially Expressed Genes

The GEO database available online: https://www.ncbi.nlm.nih.gov/geo/ (accessed on 15 June 2025) was queried with the keyword “osteoarthritis”. From the results, the human cartilage sample datasets GSE117999 (24 samples), GSE169077 (11 samples), and GSE178557 (8 samples) were selected; meanwhile, GSE114007 (38 samples) was used as an external validation dataset to verify the results. To address the substantial batch effects common across distinct datasets, we normalized the samples using the limma package in R (version 4.4.0). Principal component analysis (PCA) was then applied to the datasets both before and after normalization via the SVA package [36]. Differentially expressed genes (DEGs) were identified using a threshold of an absolute fold change > 1.5 and an adjusted *p*-value < 0.05.

### 4.2. WGCNA and Gene Screening for Cartilage Degeneration

Weighted Gene Co-expression Network Analysis (WGCNA) is a systematic bioinformatics method that uses scale-free networks to group genes into modules based on their expression correlations [37]. This approach reduces the dimensionality of complex datasets and facilitates functional exploration and trait association. We constructed a co-expression network for cartilage degeneration-related genes using the “WGCNA” package on datasets GSE117999, GSE169077, and GSE178557. The network was fitted with a soft threshold power of 10 and a scale-free topology fit index (R^2^) of 0.9. We then performed dynamic tree cutting and hierarchical clustering to generate a Topological Overlap Matrix (TOM). A gene dendrogram was built with a minimum cluster size of 50 genes [38]. Following dynamic module identification, similar modules were merged. A heatmap depicting module-trait correlations revealed the module most strongly associated with the disease phenotype. Finally, candidate genes were identified by intersecting the genes from this key module with the set of differentially expressed genes [39,40].

### 4.3. Machine Learning and Validation of Core Targets

To identify genes characteristic of cartilage degeneration, we applied three machine learning algorithms: LASSO logistic regression, SVM-RFE and RF. The LASSO algorithm, implemented with the R package “glmnet”, filtered genes by selecting the minimum λ value as the optimal predictive parameter. The optimal regularization parameter (λ) was tuned via 10-fold cross-validation, using the deviance as the loss metric. The value lambda.min, which gives the minimum cross-validated deviance, was adopted to build the final model. Genes with non-zero regression coefficients at lambda.min were retained as the LASSO-selected features. The cross-validation curve and the coefficient profile plot were generated to visualize the model selection process and internal stability [41]. SVM-RFE, an embedded feature selection method, was executed using the “e1071” package to rank genes by importance, with results validated through cross-validation to yield disease-associated characteristic genes. The dataset was split into 10 folds for cross-validation (k = 10). Within each fold, SVM-RFE iteratively removed the least important features, and the cross-validation error was computed across a range of feature subset sizes. The optimal feature set was defined as the one yielding the minimum average 10-fold CV error. The final discriminative genes were extracted accordingly, and the stability of the feature selection process was assessed by visualizing the error and accuracy curves over the feature sweep [42]. Using the “randomForest” package, an initial model was built with 500 trees, and the optimal number of trees (optionTrees) was determined as the point where the out-of-bag (OOB) error rate reached its minimum. A final RF model was then constructed with this optimal tree count. Variable importance was assessed by Mean Decrease in Gini index, and candidate diagnostic features were defined as those with an importance score > 2. Model stability was evaluated internally by the OOB error estimate [43]. The intersection of genes from these three methods defined our candidate core targets. We then used the “pROC” package to generate Receiver Operating Characteristic (ROC) curves for these hub genes and clinical features to evaluate diagnostic accuracy, and the “ggpubr” package to visualize the relative expression levels of the candidate targets [44]. Finally, we constructed an artificial neural network with the “neuralnet” and “NeuralNetTools” packages and performed logistic regression analysis using “glmnet” to assess the collective contribution of the candidate core targets to the disease [45,46].

### 4.4. Single-Gene GSEA Enrichment Analysis

Using the GO dataset and KEGG pathway gene sets as a background, we performed single-gene GSEA analysis with the “clusterProfiler” package to investigate the potential functions of RARRES1. The results were visualized using the “enrichplot” package [47].

### 4.5. Chromatography-Mass Spectrometry Analysis

The powder from four Cangxi Tongbi Capsules (Shandong Provincial Hospital of Traditional Chinese Medicine, Jinan, China) was dissolved in 100 mL of 37 °C purified water, as per the manufacturer’s instructions. One milliliter of this herbal decoction was transferred to a 2 mL volumetric flask and diluted to the mark with methanol. Following mixing, the solution underwent ultrasound-assisted extraction for 1 min; methanol was then added to compensate for any evaporation loss and to restore the volume to the mark. The mixture was centrifuged at 14,000 r/min for 10 min at 4 °C. The resulting supernatant was collected and filtered through a 0.22 μm membrane. An aliquot of the filtrate was then subjected to chromatography-mass spectrometry analysis [48]. Throughout the analysis, samples were maintained at 4 °C in the autosampler. Chromatographic separation was carried out on a Shimadzu LC-30 UHPLC system (Shimadzu, Shanghai, China) equipped with an ACQUITY UPLC^®^ HSS T3 column (2.1 × 100 mm, 1.8 µm; Waters, Milford, MA, USA). The injection volume was 12 µL, the column oven temperature was set to 40 °C, and the flow rate was 0.3 mL/min. Mobile phase A consisted of 0.1% formic acid (Millipore, Billerica, MA, USA) in water, and mobile phase B consisted of 0.1% formic acid in acetonitrile (Millipore, MA, USA). The gradient elution program was as follows: 0–2 min, 0% B; 2–3.3 min, B linearly increased from 0% to 48%; 3.3–5.1 min, B increased from 48% to 100%; 5.1–7.2 min, 100% B; 7.2–7.3 min, B decreased from 100% to 0%; 7.3–10 min, 0% B. Samples were analyzed in both positive and negative electrospray ionization (ESI) modes. After UPLC separation, mass spectrometric detection was performed on an Orbitrap Fusion mass spectrometer (Thermo Scientific, Waltham, MA, USA) equipped with a heated electrospray ionization (HESI) source. The HESI source parameters were as follows: spray voltage, +3.8 kV (positive) and −3.2 kV (negative); capillary temperature, 320 °C; sheath gas flow rate, 40 arbitrary units; auxiliary gas flow rate, 15 arbitrary units; probe heater temperature, 350 °C; and S-Lens RF level, 50. The mass spectrometric acquisition time was set to 10 min. Full MS scans were acquired over a mass range of *m*/*z* 75–1050 at a resolution of 50,000 (at *m*/*z* 200), with an automatic gain control (AGC) target of 3 × 10^6^ and a maximum injection time (max IT) of 50 ms. MS/MS spectra were acquired in a data-dependent acquisition (DDA) mode: following each full scan, the ten most intense precursor ions were selected for higher-energy collisional dissociation (HCD) fragmentation. The MS2 scans were acquired at a resolution of 7500 (at *m*/*z* 200), with an AGC target of 1 × 10^5^, a max IT of 50 ms, an isolation window of 2 *m*/*z*, and stepped normalized collision energies of 20, 30, and 40. Raw data were processed using MSDIAL software 4.1.8 for peak alignment, retention time correction, and peak area extraction. Metabolite identification was performed by accurate mass matching and MS/MS spectral library matching (mass tolerance < 0.01 Da) against an in-house database. Identification confidence was assigned at level 2 based on both MS1 and MS2 matching, according to the Metabolomics Standards Initiative (MSI) criteria.

### 4.6. Molecular Docking and Molecular Dynamics Simulations

The two-dimensional structures of all bioactive compounds were retrieved from PubChem available online: https://pubchem.ncbi.nlm.nih.gov/ (accessed on 15 June 2025) and converted into three-dimensional models using Chem3D 14.0.0.17. The three-dimensional structure of RARRES1 was generated via homology modeling using the SWISS-MODEL server, with the crystal structure of PDB entry 1WNH as the template. Subsequently, both the protein and the ligand were prepared by removing water molecules and non-essential heteroatoms, adding polar hydrogens, and assigning Gasteiger partial charges, using AutoDockTools 1.5.7. Betaine (PubChem CID: 247) was modeled in its predominant physiological zwitterionic form. The putative ligand-binding pocket was identified using the CASTp server. Molecular docking was performed with AutoDock Vina v1.2.7 using a grid box centered at (50, 25, 14) Å with dimensions of 26 × 26 × 26 Å, an exhaustiveness value of 32, and the number of binding modes (num_modes) set to 9. The final docking pose was selected based on the lowest predicted binding energy and a plausible binding orientation. MD simulations were performed using GROMACS 2022.3. The protein topology was generated using the AMBER99SB-ILDN force field, and ligand parameters were generated using the Generalized Amber Force Field (GAFF). The simulation box was sized to ensure a minimum distance of 1.0 nm between each protein atom and the box boundary. The box was filled with TIP3P water molecules and neutralized by adding Na^+^ and Cl^−^ ions. The system was energy-minimized using the steepest descent method to remove non-ideal contacts and atomic overlaps. Equilibration was performed in NVT and NPT ensembles, each for 100 ps, at 300 K and 1 bar. Production MD simulations of 100 ns were conducted under periodic boundary conditions. Temperature was controlled at 300 K using the V-rescale thermostat, and pressure was maintained at 1 bar using the Parrinello-Rahman barostat. The integration time step was 2 fs. Three independent replicate runs were carried out for each system. Long-range electrostatics were computed using the Particle Mesh Ewald (PME) method with a Fourier grid spacing of 0.16 nm. Bond lengths were constrained using the LINCS algorithm. Trajectories were visualized and analyzed using PyMOL 2.6.1. Binding free energies were calculated using gmx_mmpbsa [49,50]. After completion, the trajectory was analyzed using the software’s built-in tools to calculate the root-mean-square deviation (RMSD), the root-mean-square fluctuation (RMSF) per residue, the protein radius of gyration, and the binding free energy (MM/GBSA) [51,52].

### 4.7. Clinical Samples

This study was approved by the Ethics Committee of Nanjing Drum Tower Hospital, the Affiliated Hospital of Nanjing University Medical School. Articular cartilage samples were obtained from five patients with osteoarthritis undergoing total knee arthroplasty. From each patient, tissues were selected from both a severely damaged zone, exhibiting surface fibrillation, distinct fissures, and a cartilage defect depth exceeding 50% without exposed subchondral bone, and a relatively non-damaged zone, which appeared normal, pale white or slightly yellow, and free of obvious fissures, erosion, or ulceration. These tissue samples were then embedded in paraffin. All cartilage was harvested and processed in accordance with the Declaration of Helsinki.

### 4.8. Animal Models

The cartilage defect model is a standard tool for studying cartilage fibrosis. To assess the role of RARRES1 in cartilage repair and fibrosis, we employed this model in rats. Male Sprague-Dawley rats (8 weeks old) were obtained from Speyford Biotechnology Co., Ltd. (Beijing, China). and maintained under specific pathogen-free conditions. Using a computer-generated random number sequence, the animals were randomly allocated into four groups (*n* = 6): a Sham group, where the knee joint femoral trochlea was exposed for one minute; a Ctrl group receiving only the cartilage defect surgery; a PBS group, which received the defect surgery followed by weekly intra-articular injections of 20 µL PBS (Servicebio, Wuhan, China) beginning two weeks post-operation; and a Bet group, which received the defect surgery followed by weekly intra-articular injections of 20 µL Betamethasone (400 mM) (MCE, Shanghai, China) on the same schedule [53]. All protocols complied with animal welfare guidelines and were approved by the Ethics Committee of Drum Tower Hospital. Predefined exclusion criteria included joint infection or deformity, >20% body weight loss, and unexpected death. No animals were excluded from the study based on these criteria.

### 4.9. Histological Analysis

For morphological assessment, 5-micrometer sections were prepared from paraffin-embedded knee joint tissues [54]. The sections were deparaffinized, rehydrated, and stained with Safranin O/Fast Green and Masson’s trichrome to evaluate cartilage damage and the reparative effects of betaine. Immunofluorescence and immunohistochemistry were then used to examine the role of betaine in targeting RARRES1 to influence the chondrogenic phenotype. Following deparaffinization and rehydration, antigen retrieval was performed, and nonspecific sites were blocked with BSA PBS (Servicebio, Wuhan, China) [55]. Sections were incubated overnight at 4 °C with primary antibodies against RARRES1 (27491-1-AP, Proteintech, Wuhan, China), COL1A1 (A27820, ABclonal, Wuhan, China), and COL2A1 (A1560, ABclonal, Wuhan, China). Either fluorescent (SA00013-2, Proteintech, Wuhan, China) secondary antibodies were applied, and all subsequent steps followed standard protocols. For all in vivo and in vitro studies, each (n) represents an independent biological replicate. Three technical replicates were averaged to yield a single value per biological sample prior to statistical analysis.

### 4.10. Scanning Electron Microscopy Observation

The fixed samples were retrieved and rinsed three times for 15 min each in 0.1 M phosphate buffer (pH 7.0) (Sigma-Aldrich, Shanghai, China). They were then post-fixed in a 1% osmium tetroxide solution (Sigma-Aldrich, Shanghai, China). for 1–2 h. Following careful removal of the osmium tetroxide, the samples underwent another three 15-min rinses in the phosphate buffer. Dehydration was achieved through a graded ethanol series, with 15 min at each concentration, followed by two 20-min treatments in 100% ethanol (Sigma-Aldrich, Shanghai, China). The samples were subsequently treated with a 1:1 (*v*/*v*) mixture of ethanol and isoamyl acetate (Sigma-Aldrich, Shanghai, China) for 30 min and then with pure isoamyl acetate for one hour. Critical point drying was performed. After sputter-coating, the samples were imaged using a scanning electron microscope (JEOL Ltd., Tokyo, Japan) [56,57,58].

### 4.11. Processing of scRNA-Seq Data

This study integrated two publicly available single-cell transcriptomic datasets of OA cartilage (GSE255460 and GSE220243). Using the Seurat package, stringent quality control was first applied to the raw data, filtering out cells with a mitochondrial gene proportion exceeding 10%, cells with fewer than 200 genes detected, and genes expressed in fewer than 3 cells. Clustering analysis was performed on the filtered data using the “FindClusters” function with an appropriate resolution. Upregulated differentially expressed genes for each cell cluster were identified using the “FindAllMarkers” function. Uniform Manifold Approximation and Projection (UMAP) was employed to visualize the cell clusters. Cell annotation was performed based on highly expressed genes characteristic of each cluster. Following the annotation of cell subpopulations, the localization of RARRES1, COL2A1, COL1A1, SOX9, and ACAN across all chondrocytes was visualized on UMAP plots. To facilitate in silico gene knockout analysis, a random subset of 5000 cells was selected from the original data for subsequent analysis. The “scTenifoldKnk” package was used to construct a denoised, weighted gene regulatory network (GRN), where genes served as nodes and edge weights represented the strength of regulatory relationships between genes. For the in silico knockout, the weights of all outgoing regulatory edges from the target gene were set to zero. Subsequently, the network was projected into a low-dimensional space for visualization using a nonlinear manifold alignment algorithm. To further investigate the differentiation trajectory of the RegC subcluster, cells from this subcluster were extracted and subjected to trajectory inference and pseudotime calculation using the Monocle2 algorithm. Following the identification of differentially expressed genes associated with this trajectory, candidate genes of interest were selected for visualization of their expression levels and trajectory patterns.

### 4.12. Primary Culture of Rat Chondrocytes

Four-week-old SD rats were euthanized by cervical dislocation. The animals were immediately immersed in 75% ethanol for 5 min and then transferred to a sterile laminar flow hood. The knee joint cartilage was exposed, and cartilage tissue was harvested. The tissue was minced into small pieces and washed twice with PBS. Subsequently, the samples were initially digested with 0.25% trypsin (Servicebio, Wuhan, China) for 15 min, followed by digestion with 0.2% collagenase type II (Servicebio, Wuhan, China) in a 37 °C incubator for 2 h. After washing with PBS and centrifugation, primary chondrocytes were obtained.

### 4.13. Quantitative Polymerase Chain Reaction

Total RNA was extracted using the RNA Rapid Purification Kit (Servicebio, Wuhan, China) according to the manufacturer’s instructions. Quantitative polymerase chain reaction (qPCR) was performed by amplifying 20 μL of diluted complementary DNA (cDNA) using the SYBR Green Q-PCR Kit (Vazyme, Nanjing, China). The primer sequences for the target messenger RNA (mRNA) were as follows:

Rarres1-F: GCGTGAAGCAGTTGAAAACAGA

Rarres1-R: ACTTTTAGGGGCTTGCCAGG

β-actin-F: CCCGCGAGTACAACCTTCTTG

β-actin-R: GTCATCCATGGCGAACTGGTG

### 4.14. Surface Plasmon Resonance

Recombinant human Rarres1 protein (Biomatik, Kitchener, ON, Canada) was immobilized onto the surface of a CM5 sensor chip as the ligand. HBS-EP+ buffer (Cytiva, Shanghai, China) was employed as the running buffer. Serially diluted concentrations of betaine (0–200 μM) were used as the analyte and injected sequentially in a single-cycle kinetics mode. The association and dissociation processes were monitored in real-time at 25 °C. Data analysis was performed using Biacore T200 Evaluation Software (Cytiva, Shanghai, China), and kinetic parameters were obtained by fitting the data to a 1:1 Langmuir binding model. After each run, the chip surface was regenerated using 10 mM glycine-HCl (pH 2.0) (Cytiva, Shanghai, China).

### 4.15. Cellular Thermal Shift Assay

After heating and centrifugation, the supernatants containing soluble proteins were collected and subjected to SDS-PAGE followed by Western blotting with anti-RARRES1 and anti-GAPDH antibodies (81640-5-RR, Proteintech, Wuhan, China). GAPDH was used as a lysis control to confirm the integrity of the soluble fraction. Equal protein loading was verified by BCA (Servicebio, Wuhan, China) quantification and Ponceau S (Servicebio, Wuhan, China) staining prior to immunoblotting. The amount of soluble RARRES1 at each temperature was normalized to the signal at the lowest temperature (37 °C), which was set as 100%.

### 4.16. Reactive Oxygen Species (ROS) Detection

Cells cultured in six-well plates were incubated with H2DCFDA (SAB, Nanjing, China) working solution (10 µM) at 37 °C for 30 min in the dark. The cells were then harvested using 0.05% trypsin-EDTA solution (Epizyme, Shanghai, China), resuspended in fresh culture medium, and analyzed using a flow cytometer (BD, Franklin Lakes, NJ, USA) with excitation at 488 nm [59].

### 4.17. Protein Extraction and Western Blot Analysis

Chondrocytes cultured in six-well plates were placed on ice. A volume of 100 µL of RIPA lysis buffer (Servicebio, Wuhan, China) was added, and the lysate was thoroughly mixed. After centrifugation, the supernatant was collected, and protein concentrations were normalized using the BCA assay. Western blot analysis was performed by separating proteins via SDS-PAGE (Servicebio, Wuhan, China), followed by transfer onto a PVDF membrane. After blocking with milk, the membrane was incubated with specific primary and secondary antibodies. The gel images were converted to 8-bit grayscale using ImageJ version 1.54 (National Institutes of Health, Bethesda, MD, USA). Background subtraction was performed using the rolling ball algorithm with a radius of 50 pixels. The integrated density values of the target protein bands were measured, and the values were then normalized to the corresponding GAPDH loading control. All experiments were conducted in three independent biological replicates, and the data are presented as mean ± SD.

### 4.18. Statistical Analysis

Normally distributed data were expressed as mean ± standard deviation and compared between groups using *t*-tests. Statistical analysis was conducted via one-way analysis of variance (ANOVA) using GraphPad Prism 8. A *p*-value < 0.05 was defined as statistically significant. Normality of data distribution was assessed using the Shapiro–Wilk test, and homogeneity of variances was evaluated using Levene’s test prior to parametric analysis. For comparisons between two groups, an unpaired two-tailed Student’s *t*-test was applied when data met the assumptions of normality and equal variance; otherwise, the Mann–Whitney U test was used as a non-parametric alternative. For comparisons involving more than two groups, one-way analysis of variance (ANOVA) was performed, followed by Tukey’s honestly significant difference (HSD) post hoc test for pairwise multiple comparisons when variances were equal, or Games–Howell test when variances were unequal. A *p*-value < 0.05 was considered statistically significant. For the bioinformatics analyses, including differential expression analysis and machine learning feature selection, multiple testing correction was applied using the Benjamini–Hochberg (BH) method to control the false discovery rate (FDR). DEGs were identified using an adjusted *p*-value (FDR) < 0.05 and |log_2_ fold change| > 1.5. All statistical tests were two-sided. For each sample, three technical replicates were measured, and the mean value was used as the final determination for subsequent calculations.

## 5. Conclusions

By integrating bulk and single-cell RNA-sequencing data with bioinformatics analyses and multiple machine-learning algorithms, this study systematically screened and identified RARRES1 as a key gene associated with cartilage degeneration. Using an ex vivo chondrocyte fibrosis model and a rat cartilage defect repair model in vivo, we further validated the marked upregulation of RARRES1 in fibrotic cartilage and elucidated the molecular mechanism by which betaine alleviates fibrosis and promotes hyaline cartilage repair—specifically, through suppressing RARRES1, upregulating RGS2, and facilitating reactive oxygen species clearance. Collectively, these findings establish, for the first time, that RARRES1 is associated with altered RGS2 expression as a critical mediator in cartilage fibrosis, offering novel pathophysiological insights into osteoarthritis and providing a theoretical foundation for early diagnostic and therapeutic strategies targeting RARRES1.

## Data Availability

The datasets generated or analysed during the current study are available in the GEO database or in the Supplementary Materials.

## Supplementary Materials

The following supporting information can be downloaded at: https://www.mdpi.com/article/10.3390/ijms27156684/s1.

## Abbreviations

ACAN	Aggrecan
APOD	Apolipoprotein D
Bet	Betaine
C10orf10	Chromosome 10 open reading frame 10
COL1A1	Collagen Type I Alpha 1 Chain
COL2A1	Collagen Type II Alpha 1 Chain
CSN1S1	Alpha-S1-Casein
CTGF	Connective Tissue Growth Factor
DEGs	Differentially Expressed Genes
ECM	Extracellular Matrix
FC	Fibrocartilage
GEO	Gene Expression Omnibus
GO	Gene Ontology
GSEA	Gene Set Enrichment Analysis
ICRS	International Cartilage Repair Society
KEGG	Kyoto Encyclopedia of Genes and Genomes
LASSO	Least Absolute Shrinkage and Selection Operator
MMP3	Matrix Metalloproteinase 3
OA	Osteoarthritis
PBS	Phosphate Buffered Saline
qPCR	Quantitative Polymerase Chain Reaction
RARRES1	Recombinant Retinoic Acid Receptor Responder 1
RegC	Regulatory Chondrocyte
RF	RandomForest
RGS2	Regulator of G Protein Signaling 2
ROS	Reactive Oxygen Species
SOX9	SRY-box Transcription Factor 9
SVM-RFE	Support Vector Machine-Recursive Feature Elimination
UMAP	Uniform Manifold Approximation and Projection
VEGFA	Vascular Endothelial Growth Factor A
WB	Western Blot
WGCNA	Weighted Gene Co-expression Network Analysis
