# Supplementary material for: Betaine Downregulates RARRES1 to Alleviate Cartilage Fibrosis and Promote Hyaline Cartilage Repair
Source: Int J Mol Sci. 2026 Jul 27;27(15):6684. doi: 10.3390/ijms27156684 (PMC13467245; doi:10.3390/ijms27156684)
Supplement: Supplementary file 1 [file ijms-27-06684-s001.zip › Supplementary Figure.pdf]

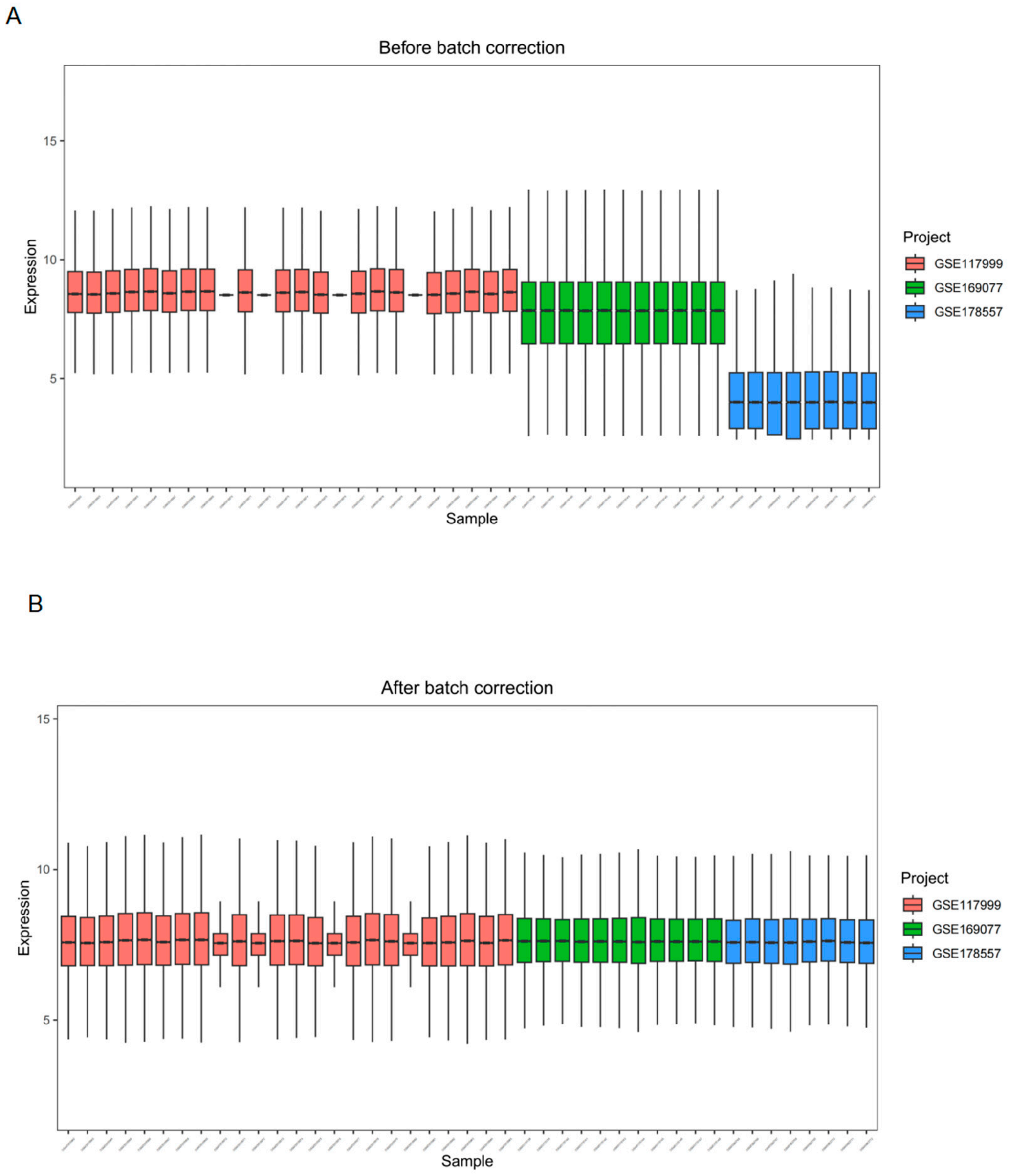

Supplementary Figure S1. Comparison of data before and after normalization. Gene expression profiles before (A) and after (B) batch correction. These results indicate that batch correction effectively improves comparability across datasets.

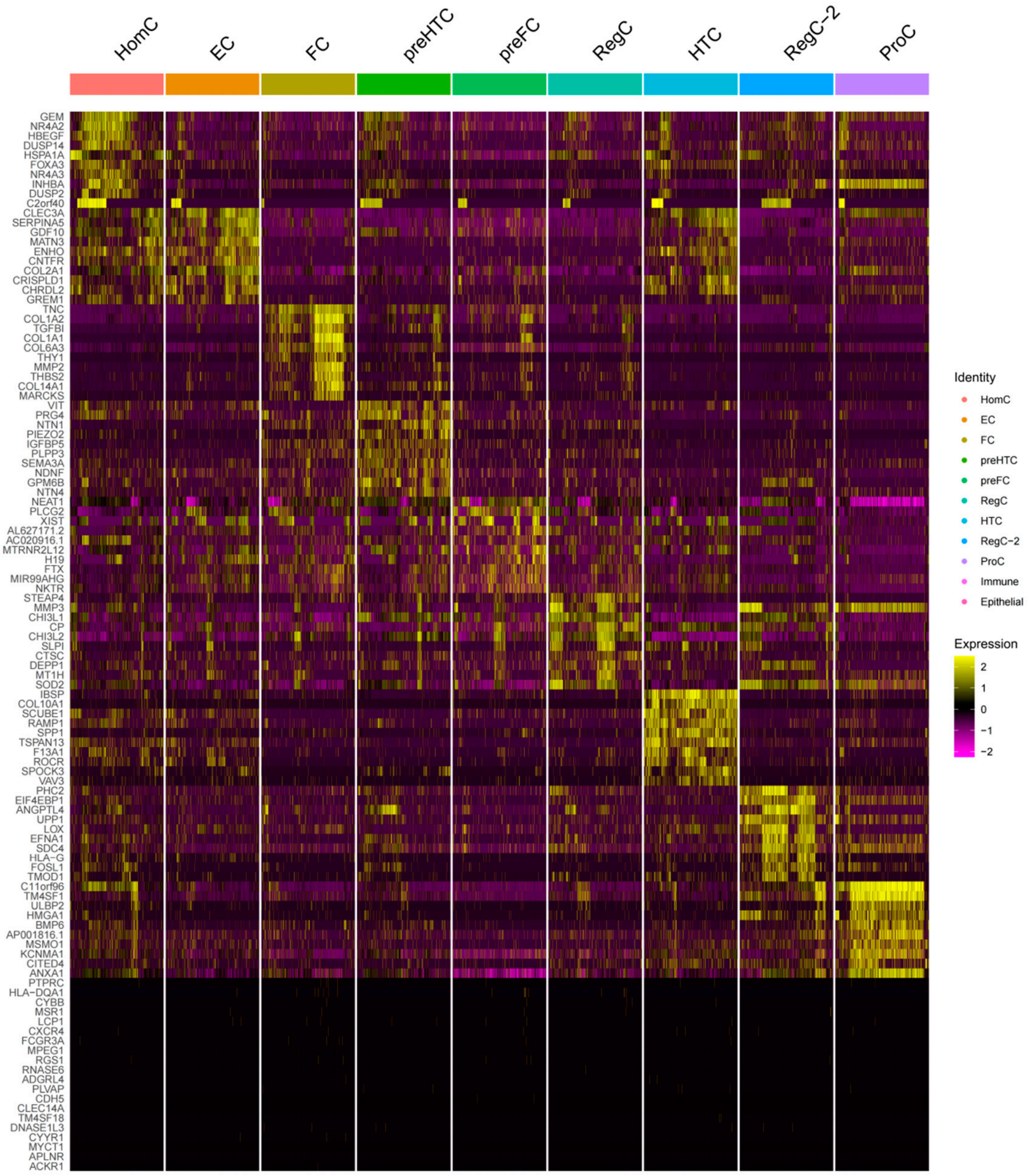

Supplementary Figure S2. Heatmap illustrating the annotation of cartilage single-cell subpopulations.

The x-axis denotes the cartilage subpopulations, and the y-axis indicates the signature genes.

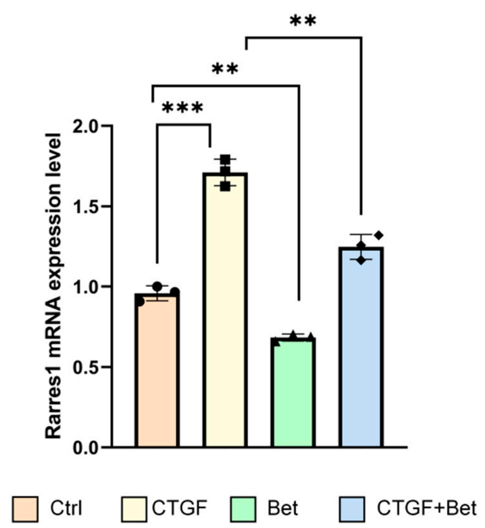

Supplementary Figure S3. Betaine inhibits the transcription level of Rarres1.
qPCR results demonstrated that CTGF induced the upregulation of Rarres1 mRNA, whereas betaine reduced the level of Rarres1 mRNA in chondrocytes, and this inhibitory effect effectively attenuated the CTGF-induced increase in Rarres1 mRNA.

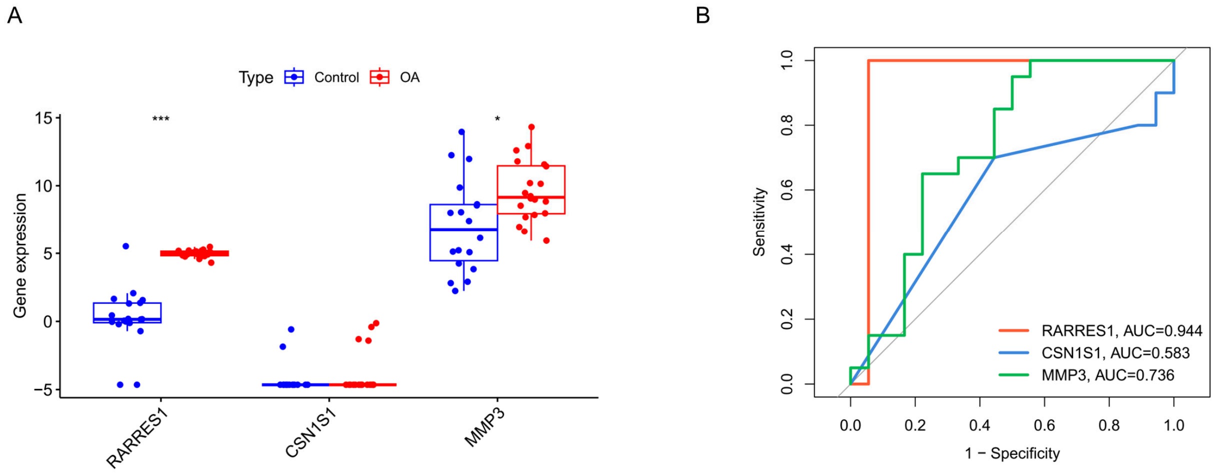

Supplementary Figure S4. External dataset validation of RARRES1, CSN1S1 and MMP3.
(A) Box plots showing the expression levels of RARRES1, CSN1S1, and MMP3 in the validation cohort; (B) ROC curves and the corresponding AUC values for RARRES1, CSN1S1, and MMP3 in the validation cohort.

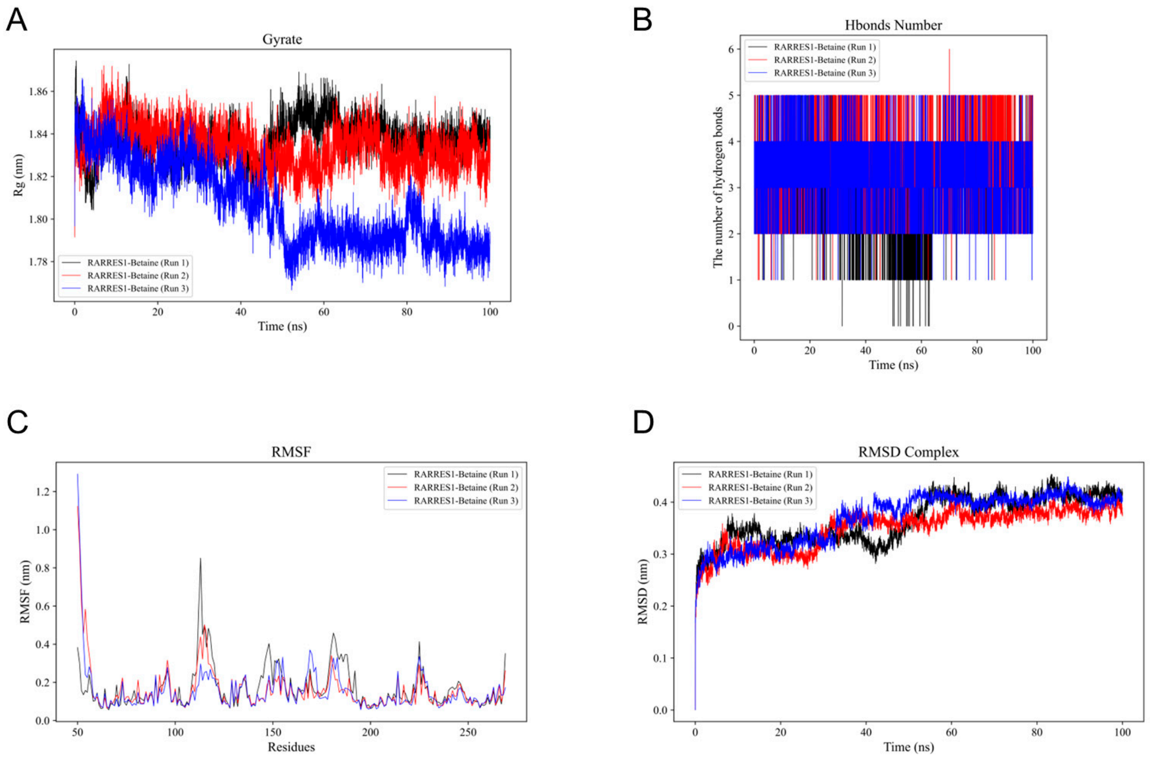

Supplementary Figure S5. Molecular Dynamics Simulation Outcomes for Betaine in Complex with RARRES1.

(A) Radius of gyration (Rg) plots for three replicate simulations of the RARRES1–betaine complex. The Rg values remained stable, with averages of 1.84 ± 0.01 nm (Run 1), 1.83 ± 0.01 nm (Run 2), and 1.81 ± 0.02 nm (Run 3);

(B) Hydrogen-bond number plots for the same replicate simulations. The average counts of intermolecular hydrogen bonds between RARRES1 and betaine were 2.91 ± 0.79 (Run 1), 3.44 ± 0.71 (Run 2), and 3.17 ± 0.60 (Run 3);

(C) Root mean square fluctuation (RMSF) plots for the three replicate simulations. The average RMSF values of RARRES1 residues were 0.17 ± 0.11 nm (Run 1), 0.16 ± 0.12 nm (Run 2), and 0.16 ± 0.12 nm (Run 3);

(D) Root mean square deviation (RMSD) plots for the three replicate simulations. The overall RMSD values of the complex were 0.37 ± 0.04 nm (Run 1), 0.35 ± 0.04 nm (Run 2), and 0.37 ± 0.05 nm (Run 3).

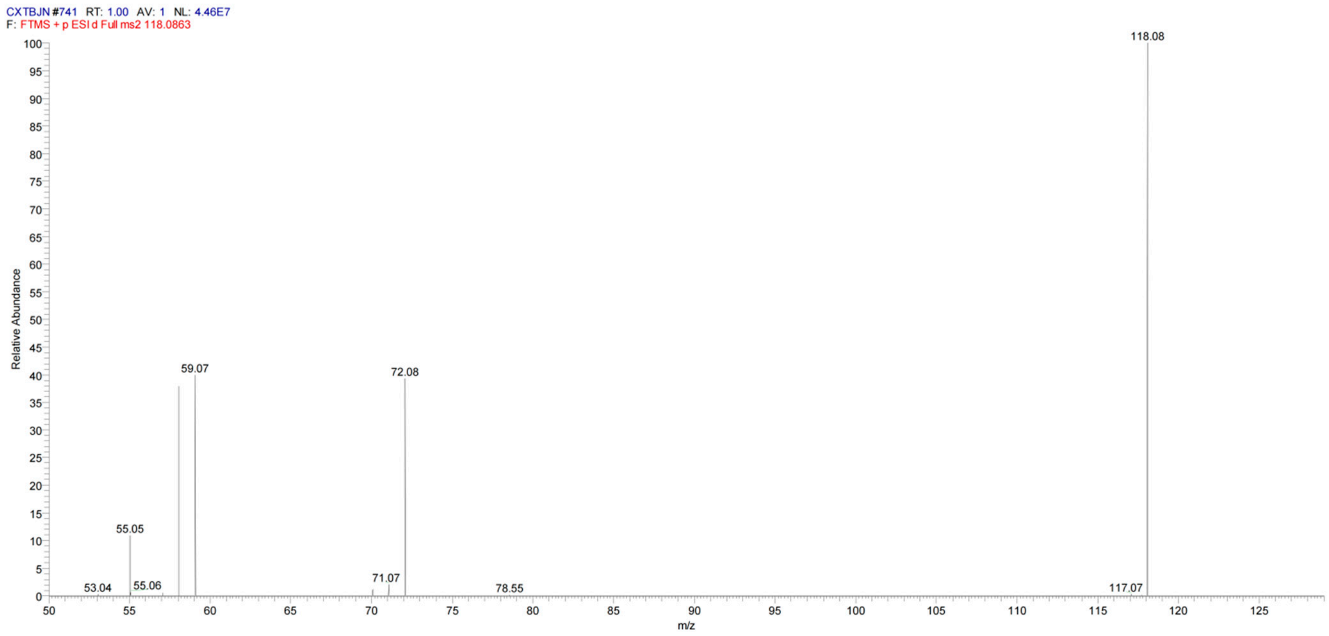

Supplementary Figure S6. Characterization of Characteristic Fragment Ions of Betaine by Tandem Mass Spectrometry.

Characteristic ion pair of betaine: 118.08 > 59.07.
